# Deficits in cortical, diencephalic and midbrain gray matter in alcoholism measured by VBM: Effects of co-morbid substance abuse^☆^^☆☆^^★^

**DOI:** 10.1016/j.nicl.2013.03.013

**Published:** 2013-04-01

**Authors:** Erica N. Grodin, Henry Lin, Caitlin A. Durkee, Daniel W. Hommer, Reza Momenan

**Affiliations:** Laboratory of Clinical Studies, National Institute on Alcohol Abuse Alcoholism, National Institutes of Health, Bethesda, MD 20892-7003, USA

**Keywords:** Voxel-based morphometry, Alcoholism, Polysubstance use, Gray matter structure

## Abstract

**Objective:**

Alcoholism has been associated with a widespread pattern of gray matter atrophy. This study sought to investigate the spectrum of volume alterations in a population of alcoholics with only alcohol dependence, polysubstance abusing alcoholics, and a comparison population of healthy controls.

**Method:**

Thirty-seven ‘pure’ alcoholics, 93 polysubstance abusing alcoholics, and 69 healthy controls underwent structural T1 MRI scans. Voxel-based morphometry was performed to investigate gray matter alterations.

**Results:**

Alcoholic dependent inpatients (both with and without a history of DSM-IV substance abuse/dependence diagnosis) displayed significant gray matter differences in the mesial region of the frontal lobe and right temporal lobe. ‘Pure’ alcoholics exhibited a pattern of subcortical changes similar to that seen in Wernicke–Korsakoff Syndrome when compared to polysubstance abusing alcoholics. ‘Pure’ alcoholics and polysubstance abusing alcoholics did not differ significantly in measures of cortical gray matter, liver function, or nutrition.

**Conclusions:**

These findings reinforce the accepted literature in regards to frontal lobe gray matter atrophy in alcohol dependence. This study calls for additional research in order to investigate the spectrum from uncomplicated alcoholism to Wernicke–Korsakoff Syndrome. Further research is needed to elucidate the exact cause of this pattern of differences and to determine what factors are responsible for the patterns of gray matter reduction or difference in ‘pure’ and polysubstance abusing alcoholics.

## Highlights

• Large scale voxel-based morphometry study on alcoholics and healthy controls.
• Alcoholics display less gray matter in the mesial frontal lobe and the temporal lobe.
• “Pure” alcoholics show a pattern of deficit similar to that of Wernicke's Encephalopathy.

## 1 Introduction

It has been established that the heavy consumption of alcohol due to alcohol dependence causes gray matter loss (Bjork et al., 2003, Fein et al., 2002, Jernigan et al., 1991). Postmortem studies have demonstrated a lower mean weight in the brains of alcoholics without any other neurological disease (Harper and Blumbergs, 1982), as well as brain tissue loss in chronic alcoholics (Harper and Kril, 1985). Several studies have found that alcohol dependent individuals have specific regional volume loss, particularly in the frontal lobe (Harper and Matsumoto, 2005, Moselhy et al., 2001, Pfefferbaum et al., 1997).

Many magnetic resonance imaging studies of alcoholism have focused primarily on the cerebrum and to a lesser extent, the cerebellum. Recently investigators have begun using voxel-based morphometry (VBM) (Ashburner and Friston, 2000) to evaluate gray matter differences. This procedure also allows for the global analysis of brain structures, including the diencephalon and midbrain, as well as the cerebrum. VBM has been used successfully in the investigation of schizophrenia (Salgado-Pineda et al., 2011), aging (Good et al., 2001), and Alzheimer's disease (Derflinger et al., 2011).

VBM has also been used to investigate brain morphometrical differences in alcoholics relative to healthy controls. (Mechtcheriakov et al., 2007, Rando et al., 2011). One study investigated both white and gray matter loss in 22 alcohol dependent patients and in age and sex matched healthy controls. The alcoholic patients displayed an overall decrease in the gray matter volume in the thalamus, posterior hippocampus, and frontal cortical areas, as well as white matter atrophy in the pons and the cerebellum. The other study compared the gray matter volumes of 45 abstinent alcohol-dependent patients and 50 healthy control subjects. The alcohol dependent patients displayed gray matter volume loss in the lateral prefrontal cortex, the medial frontal cortex, and the posterior cingulate gyrus.

Gray matter alterations have also been investigated in other populations of substance abusers, including cocaine dependent patients (Franklin et al., 2002), heavy cannabis users (Cousijn et al., 2012), methamphetamine abusers (Schwartz et al., 2010), and heroin dependent subjects (Liu et al., 2009). Very few studies have examined morphometrical alterations in polysubstance abusers (Liu et al., 1998, Reid et al., 2008, Tanabe et al., 2009). Liu and colleagues found that polysubstance abusers had smaller prefrontal lobes bilaterally relative to healthy controls. This volume loss was found only in gray matter. Tanabe and colleagues also found reduced gray matter volume in the frontal lobe of substance dependent individuals, specifically in the medial orbital frontal cortex.

Several years ago we compared forebrain volumes among inpatients at the National Institutes of Health (NIH) Clinical Center alcoholism treatment unit with or without co-morbid abuse of substances in addition to alcohol (Bjork et al., 2003). We found very little difference in brain volume between alcoholic subjects with or without co-morbid substance abuse. However, this study only measured overall forebrain volumes of gray and white matter and did not investigate diencephalon or midbrain alterations, regions which have been noted to be affected in alcoholism (Zuccoli et al., 2007). In addition, regional differences in brain volume were not examined in our earlier study.

In this report we used a VBM approach to compare regional brain volume differences between individuals with alcohol dependence and controls. We hypothesized that the alcohol dependent population would display less gray matter in the frontal lobes, as well as in the cerebellar cortex. Additionally we sought to investigate, we believe for the first time, regional differences between polysubstance abusing alcoholics and alcoholics with only alcohol dependence. Since our previous work comparing overall gray matter forebrain volumes in these groups did not find a significant difference (Bjork et al., 2003), we tested the hypothesis that the groups would differ in regional gray matter volume, but made no specific hypothesis about the direction of the difference.

## 2 Method

All recruitment and testing procedures were reviewed and approved by the National Institute on Alcohol Abuse and Alcoholism (NIAAA) Institutional Review Board. After complete detoxification and withdrawal, experimental procedures (psychometric interviews and magnetic resonance imaging) were explained, and all patients provided written informed consent to participate.

### 2.1 Subjects

Subjects with alcohol dependence (N = 130: 93 Caucasian, 34 African American, 1 Hispanic, 1 Asian, 1 Other; 47 Female), ages 20–64, were admitted for an inpatient alcoholism treatment program. Community-recruited subjects (N = 69: 47 Caucasian, 12 African American, 4 Hispanic, 4 Asian, 2 East Indian; 22 Female), ages 19–63, with no history of significant medical illness or psychiatric disorders were also included for comparison. Alcohol-dependent patients whose estimated IQ was below 80, who had neurological abnormalities, who had a history of psychotic symptoms, or who were not eligible for an MRI scan were not included in the sample. All subjects were assessed with the Structured Clinical Interview for DSM-IV, which determined that each inpatient met criteria for alcohol dependence and that the comparison subjects did not meet DSM-IV criteria for current axis I disorders. A urine sample was collected to verify drug abstinence. All participants received a physical examination to ensure good general physical and neurological health. A social worker administered a semi-structured lifetime drinking history interview to each subject. Alcohol use history was divided into epochs of various use patterns according to each respondent's history, and from these epochs we calculated three drinking history parameters: 1) age at onset of heavy drinking, defined a priori as the age at which the subject reported first consuming the equivalent of 90 drinks in a 1-month period; 2) years of heavy drinking, defined as the cumulative total contiguous or noncontiguous months during which the subject drank 90 drinks per month (note: since subjects often maintain this high a level of alcohol use for at least 12 consecutive months, months are summed into years); and 3) estimated lifetime alcohol consumption (in kg), which is a summation of all alcohol ingestion, including during periods where ingestion did not reach 90 drinks per month. Age, average years of heavy drinking, years of education, lifetime alcohol consumption, age of onset of alcoholism, past Axis I diagnoses, BMI, gray matter, and intracranial volumes can be found in Table 1.

**Table 1:** Demographics.

Characteristic of participants	‘Pure’ alcoholics N = 37		Polysubstance abusing alcoholics N = 93		Healthy controls N = 69	
	Mean	SD	Mean	SD	Mean	SD
Male/female	21/16	–	62/31	–	47/22	–
Age at admission	40.2	9.2	38.1	7.1	36.6	1.1
Years of education^a^	14.5	2.6	13.2	2.3	16.8	0.3
Years of heavy drinking	10.3	7.5	11.9	7.1	–	–
Lifetime alcohol consumption (kg)^a^	467.0	470.6	583.0	458.3	10.1	16.1
Age of alcoholism onset	25.3	9.5	22.7	6.9	–	–
BMI	26.9	4.4	25.8	4.5	27.49	1.3
Intracranial volume (ml)	1323.6	119.7	1307.6	150.9	1353.5	146.5
Gray matter (ml)^a^	553.2	57.4	560.3	63.3	587.3	55.6

*Drug abuse/dependence*						
Cocaine dependence	0	–	50	–	0	–
Cocaine abuse	0	–	16	–	0	–
Cannabis dependence	0	–	50	–	0	–
Cannabis abuse	0	–	13	–	0	–
Opioid dependence	0	–	14	–	0	–
Opioid abuse	0	–	6	–	0	–
Sedative dependence	0	–	14	–	0	–
Sedative abuse	0	–	7	–	0	–
Amphetamine dependence	0	–	10	–	0	–
Amphetamine abuse	0	–	4	–	0	–
Hallucinogen/PCP dependence	0	–	17	–	0	–
Hallucinogen/PCP abuse	0	–	18	–	0	–
“Other” substance abuse	0	–	2	–	0	–

*Past Axis I disorders*						
Major depression	25	–	70	–	0	–
Mood disorder	25	–	72	–	0	–
Anxiety disorder	15	–	54	–	0	–
Eating disorder	1	–	6	–	0	–
Post-traumatic stress disorder	12	–	33	–	1	–
Attention deficit hyperactive disorder	5	–	28	–	1	–
Obsessive compulsive disorder	10	–	36	–	0	–
Conduct disorder	6	–	29	–	0	–

Because most polysubstance abusing alcoholics used more than one substance in addition to alcohol the total diagnoses add up to more than 93.

a Significant difference between all alcoholics and controls at p < 0.05.

### 2.2 Cognitive tests

A selective reminding task used in the current study has been described previously (Weingartner et al., 1996). In short, a list of 12 words is read to the patient and the patient is asked to recall as much as possible. Then the patient is reminded of the words they did not remember. This procedure is repeated 8 times. This procedure, the Buschke selective reminding task (Buschke and Fuld, 1974), has been widely used to measure memory impairments in dementia, head injury, aging, child development, drugs and therapies (for a review, see (Kraemer et al., 1983)). We used total trials required to learn the word list as a measure of episodic memory function.

Intelligence was estimated by two subtests of the Wechsler Adult Intelligence Scale—Revised (WAIS-R; (Wechsler, 1981)), Vocabulary and Block Design (see Table 1). These two subtests have previously been used as a “short-form” of the WAIS-R to estimate IQ, with reasonably good results (Silverstein, 1982, Silverstein, 1983). Separately, they provide rough estimates of verbal or “crystallized” intelligence, and nonverbal or “fluid” intelligence, respectively. In the standardization sample, Vocabulary highly correlated with Verbal IQ, while Block Design highly correlated with Performance IQ (Wechsler, 1981).

### 2.3 MRI acquisition

All subjects were scanned using a 1.5 T General Electric MRI scanner (General Electric, Milwaukee, WI) and a standard head coil. Whole-brain high-resolution coronal structural scans were collected using a T1-weighted magnetization-prepared rapid gradient echo (MPRAGE) pulse sequence with matrix 256 × 256 × 124, repetition time (TR) = 100 ms, echo time (TE) = 12 ms, field of view (FOV) = 24 cm, and voxel size of (0.9375 × 0.9375 × 2.0) mm^3^. Alcoholic subjects were scanned 21.5 ± 5.3 days from admission date and thus had been free of alcohol or other substance use for at least that duration.

### 2.4 Voxel-based morphometry

Structural data was analyzed with FSL-VBM, version 1.1, a gray matter voxel-based morphometry analysis (Ashburner and Friston, 2000) software carried out with FSL tools (Smith et al., 2004). First, structural images were brain-extracted using BET (Smith, 2002). Next, tissue-type segmentation was carried out using FAST4 (Zhang et al., 2001).The resulting gray matter partial volume images were then aligned to MNI152 standard space using the affine registration tool FLIRT (Jenkinson et al., 2002), followed by nonlinear registration using FNIRT (Andersson et al., 2007a, Andersson et al., 2007b), which uses a b-spline representation of the registration warp field (Rueckert et al., 1998). The resulting images were averaged to create a study-specific template (with equal number of patient and control subjects to avoid bias), to which the native gray matter images were then non-linearly re-registered. The registered partial volume images were then modulated (to correct for local expansion or contraction) by dividing by the Jacobian of the warp field. The modulated segmented images were then smoothed with an isotropic Gaussian kernel with a sigma of 3 mm. Finally, voxelwise GLM was applied using permutation-based non-parametric testing, correcting for multiple comparisons across space. Ten thousand permutations were run for each comparison. In all of the comparisons age and years of education were included as covariates. This approach is unbiased, in that it requires no a priori information about the location of these possible differences in the gray matter, and is not operator-dependent. It follows the optimized VBM protocol developed by Good et al. (2001). Therefore, no additional corrections are needed. All using this method are reported for at a significance level p < 0.01.

## 3 Results

The following comparisons were made using the above VBM procedure: alcoholic inpatients vs. healthy controls, alcoholics with only alcohol dependence vs. alcoholic polysubstance abusers, alcoholics with only alcohol dependence vs. healthy controls, and alcoholic polysubstance abusers vs. healthy controls. The polysubstance users included all inpatient alcoholics who reported past or present substance abuse or substance dependence and met DSM IV criteria for past or current substance abuse or dependence. Only 28.5% of the inpatient alcoholics did not meet DSM IV criteria for a substance abuse disorder other than alcoholism at some time in their life. In addition, most of the alcoholics who met criteria for substance abuse other than alcohol abuse, abused more than one other substance. Thirty-four inpatients met criteria for alcohol dependence and one additional substance use disorder. Out of those 34, 14 alcohol dependent subjects met criteria for cannabis abuse or dependence, 16 alcohol dependent subjects met criteria for cocaine abuse or dependence, 3 alcohol dependent subjects met criteria for hallucinogen/PCP abuse or dependence, and 1 alcoholic dependent subject met criteria for sedative dependence. The breakdown of substance use diagnoses can be found in Table 1. Because of the small number of subjects in each individual substance abuse category, all polysubstance abusing and dependent alcoholics were grouped together. In all of the comparisons age and years of education were included as covariates. In the alcoholics with only alcohol dependence (‘pure’ alcoholics) vs. polysubstance abusers comparison, years of heavy drinking was also included as a covariate. Due to a large number of alcoholics who were also smokers (N = 107), an alcoholic smoker vs. non-smoker comparison (N = 23) was run to ensure that smoking was not acting as a confounding variable in the previous analyses.

### 3.1 All alcoholic inpatients vs. healthy controls

**Fig. 1:**
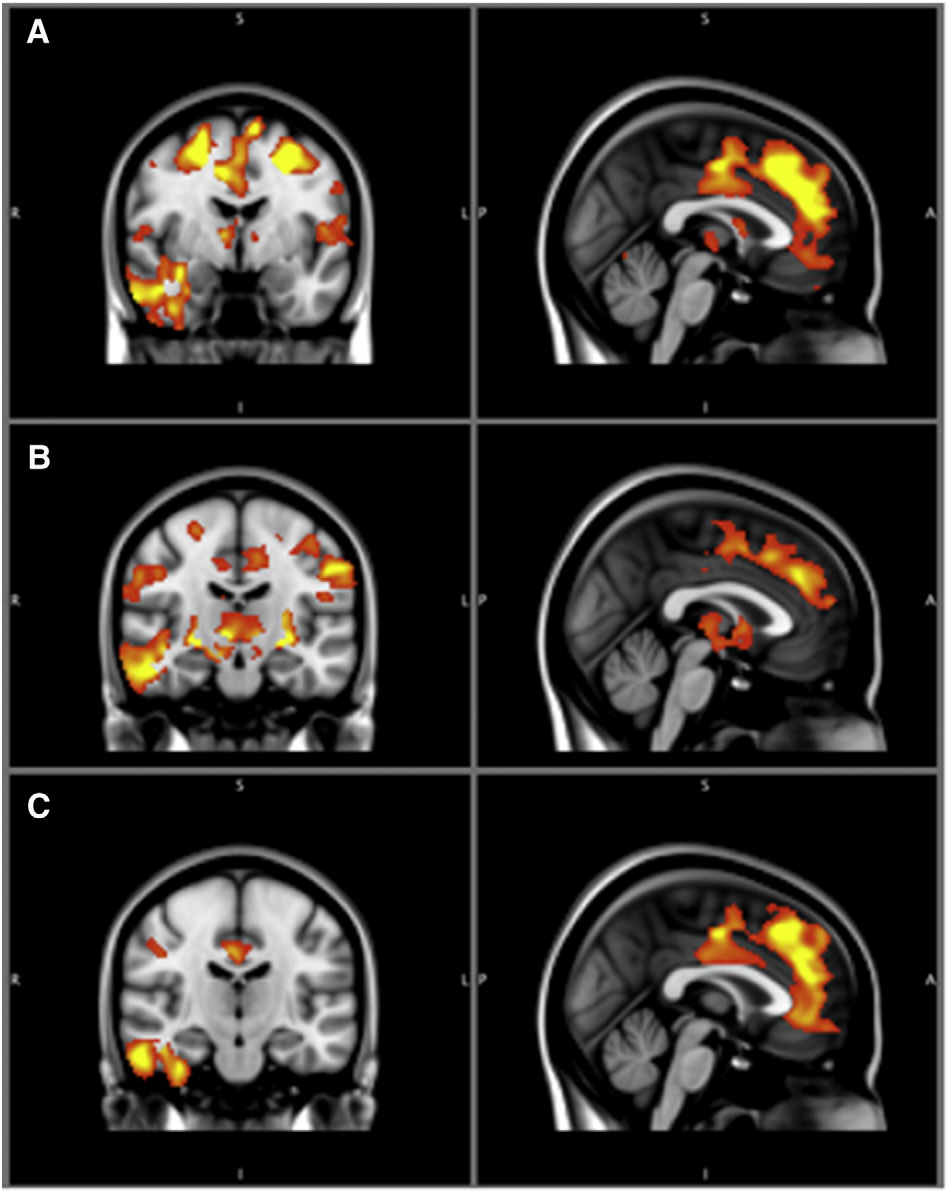
All healthy control comparisons. The color bar represents the t-value from 2.3 to 4 for significantly different clusters. Panel A displays the gray matter alterations that the full alcoholic group exhibits relative to healthy controls. Panel B shows the differences that the ‘pure’ alcoholics display relative to healthy controls. Panel C displays the alterations that the polysubstance abusing alcoholics display relative to healthy controls.

Alcoholics had significant gray matter differences in many frontal regions, such as the medial surface of the superior frontal gyrus, right and left middle frontal gyri, right and left inferior frontal gyri, and left frontal pole, as well as in the right temporal lobe, right thalamus, and right hippocampus (see Table 2, Fig. 1A).

**Table 2:** VBM results.

Comparison	Brain region	Milliliters	X	Y	Z	P-value (corrected)
HC > ADS	Medial frontal gyrus	28.9	0	24	46	< 0.001
	Right inferior temporal gyrus	14.6	62	− 26	− 26	< 0.001
	Superior frontal gyrus	7.7	− 22	6	52	< 0.001
	Cingulate gyrus	6.1	14	− 8	44	< 0.001
	Frontal pole	5.0	42	52	16	< 0.001
	Inferior frontal gyrus	4.0	− 52	10	24	< 0.001
	Left hippocampus	3.4	− 12	− 44	10	< 0.001
	Angular gyrus	3.2	− 48	− 54	34	< 0.001
	Frontal pole	2.7	− 28	48	14	< 0.001
	Lingual gyrus	2.4	14	− 42	− 10	< 0.001
	Postcentral gyrus	1.8	− 56	− 16	36	< 0.001
	Precuneus	1.7	18	− 68	30	< 0.005
	Inferior frontal gyrus	1.4	54	12	22	< 0.005
	Right thalamus	.77	8	− 12	− 2	< 0.01
	Right amygdala	.70	16	− 14	− 12	< 0.01
HC > ‘Pure’	Precuneus cortex	9.12	18	− 56	14	< 0.001
	Right Inferior temporal gyrus	8.392	62	− 26	− 24	< 0.001
	Superior frontal gyrus	6.384	18	40	44	< 0.001
	Right thalamus	5.152	20	− 26	4	< 0.001
	Precentral gyrus	4.632	24	− 6	60	< 0.001
	Angular gyrus	4.496	− 50	56	38	< 0.001
	Middle frontal gyrus	4.168	− 28	− 2	48	< 0.001
	Inferior frontal gyrus	2.6	− 52	10	24	< 0.001
	Frontal pole	2.512	− 24	50	10	< 0.001
	Lateral occipital cortex	2.376	42	− 60	32	< 0.001
	Superior parietal lobule	1.704	− 16	− 56	58	< 0.001
	Intracalcarine cortex	1.448	− 22	− 70	6	< 0.001
	Paracingulate gyrus	1.296	0	36	32	< 0.001
	Postcentral gyrus	1.224	26	− 32	50	< 0.001
	Left thalamus	1.224	− 8	− 16	− 2	< 0.001
	Cingulate gyrus	1.168	14	− 6	46	< 0.001
	Left putamen	1.12	− 26	− 14	− 6	< 0.005
	Right putamen	1	26	− 16	− 6	< 0.005
HC > Poly	Superior frontal gyrus — medial region	15.464	0	24	46	< 0.001
	Right inferior temporal gyrus	9.2	62	− 8	− 32	< 0.001
	Middle frontal gyrus	7.656	34	20	52	< 0.001
	Superior frontal gyrus	6.192	− 28	22	50	< 0.001
	Right temporal pole	2.808	38	10	− 36	< 0.001
	Cingulate gyrus	2.408	0	− 10	24	< 0.001
	Precentral gyrus	1.984	− 44	4	24	< 0.001
Poly > ‘Pure’	Thalamus (bilateral)	1.976	6	− 16	16	< 0.001
	Parahippocampal gyrus	1.712	20	− 26	− 24	< 0.001
	Brain stem	1.04	− 10	− 26	− 16	< 0.005

All coordinates are presented in MNI space. Comparisons: Healthy controls vs. All Alcoholics (HC > ADS) — alcoholics less gray matter relative to controls. Total volume of significantly different cortex was 82.9 ml. Total volume of significantly different subcortex was 1.47 ml. Healthy controls vs. ‘Pure’ Alcoholics (HC > ‘Pure’) — areas in which alcoholics with only alcohol dependence display less gray matter relative to healthy controls. Total volume of significantly different cortex was 51.51 ml. Total volume of significantly different subcortex was 8.49 ml. Healthy controls vs. Polysubstance abusing alcoholics (HC > Poly) — areas in which alcoholics with polysubstance abuse or dependence display less gray matter relative to healthy controls. Total volume of significantly different cortex was 45.71 ml. Total volume of significantly different subcortex was 0 ml. Polysubstance abusing alcoholics vs. ‘Pure’ Alcoholics (Poly > ‘Pure’) — areas in which alcoholics with only alcohol dependence display less gray matter relative to alcoholics who are polysubstance abusers. The volume of significantly different tissue was 4.7 ml, the majority being subcortical.

### 3.2 ‘Pure’ alcoholics vs. healthy controls

Alcoholics who were diagnosed with only alcohol dependence displayed similar gray matter alterations as the entire alcoholic inpatient sample relative to healthy control comparison. Areas that displayed gray matter volume differences included: the superior, middle, and inferior frontal gyri, frontal pole, bilateral thalamus, bilateral putamen, right inferior temporal gyrus, and the cingulate gyrus (see Table 2, Fig. 1B).

### 3.3 Alcoholics with polysubstance abuse vs. healthy controls

Polysubstance abusing alcoholics displayed significant gray matter alterations in the mesial region of the frontal lobe, including superior frontal, cingulate and para-cingulate gyri. On the lateral surface of the brain the middle and superior frontal gyri and the right inferior temporal gyrus were affected. No regions of significant gray matter volume difference were found in the diencephalon or below (see Table 2, Fig. 1C).

### 3.4 ‘Pure’ alcoholics vs. alcoholics with comorbid substance abuse/dependence

**Fig. 2:**
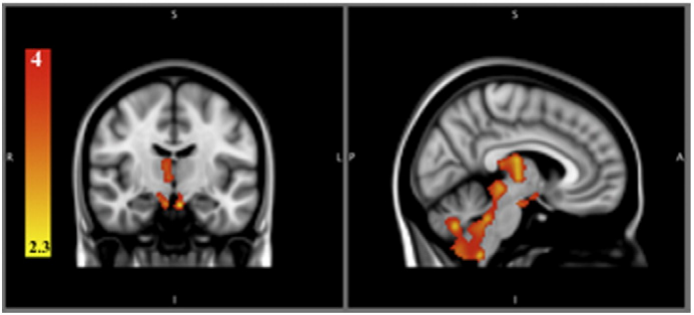
Polysubstance abusing alcoholics vs. ‘pure’ alcoholics. The color bar represents the t-value from 2.3 to 4. Significant clusters of neurodegeneration in ‘pure’ alcoholics relative to polysubstance abusing alcoholics.

‘Pure’ alcoholics showed significant gray matter differences relative to alcoholics who were diagnosed with additional comorbid substance abuse or dependence in the following areas: thalamus, brain stem, mammillary bodies, and cerebellum (see Table 2, Fig. 2).

In order to rule out the potential compensatory effect of pro-inflammation due to stimulant abuse in gray matter differences, we conducted three additional VBM analyses among polysubstance abusing alcoholics with stimulant abuse and dependence and those without. First, in an overall comparison of subjects with cocaine (and other comorbid uses) (n = 66) with those polysubstance abusing alcoholics without cocaine use (n = 27), no significant VBM differences were observed. Second, we compared cocaine-only users (n = 16) relative to all other polysubstance users (n = 77), and found the latter group to have greater gray matter volume in the right thalamus, right brainstem, and cerebellum — similar areas to those found in the comparison between polysubstance abusers and ‘pure’ alcoholics. Third, in a follow-up analysis we removed the amphetamine/stimulant users from the other polysubstance group (n = 22) and no group differences were observed between this group and cocaine-only users.

In an effort to rule out the effect of past psychological disorders as the cause of the differences between ‘pure’ alcoholics and the alcoholics with polysubstance abuse, we ran similar VBM analysis on alcoholic subjects with comorbid Axis I diagnoses. In doing so, we performed VBM analyses between all alcoholic subjects with anxiety vs. those without anxiety; all alcoholic subjects with depression vs. all alcoholic subjects without depression; and all alcoholic subjects with PTSD vs. all alcoholic subjects without PTSD. If any of these comorbidities was the cause of above results between ‘pure’ and poly substance abuser alcoholics, we should have detected overlapping regions of difference. This overlap did not occur in any of the comparisons (see Table 4).

**Table 4:** Psych results.

Comparison	Brain region	Milliliters	X	Y	Z	P-value (corrected)
Anxiety > NoAnxiety	Medial–posterior cerebellum	5.6	2	− 68	− 26	< 0.001
	Left culmen	4.0	− 28	− 28	− 32	< 0.001
	Right inferior MPFC	2.78	18	28	− 20	< 0.001
	Right superior temporal gyrus	2.17	36	− 4	− 18	< 0.001
	Right parahippocampal gyrus	2.03	18	− 28	− 4	< 0.001
	Right caudate	1.21	12	28	− 2	< 0.001
	Left superior temporal gyrus	0.92	− 32	6	− 16	< 0.005
Depression > NoDepression	Right lingual gyrus Right fusiform gyrus	5.49 1.10	6 − 36	− 82 − 60	− 16 − 20	< 0.001 < 0.005
	Right vermis	0.72	− 10	− 68	− 36	< 0.01
PTSD > NoPTSD	Left declive	3.14	− 2	− 72	− 12	< 0.001
	Left lingual gyrus	0.84	− 14	− 88	− 2	< 0.005

All coordinates are presented in MNI space.

Anxiety > NoAnxiety, regions in which alcoholics without anxiety demonstrated less gray matter relative to alcoholics with anxiety. Depression > NoDepression — regions in which alcoholics without depression demonstrated less gray matter than alcoholics with depression. PTSD > NoPTSD — regions in which alcoholics without PTSD demonstrated less gray matter than alcoholics with PTSD. There was no gray matter differences in the opposite direction to the above mentioned.

### 3.5 Alcoholic smokers vs. alcoholic non-smokers

The only area of possible significant gray matter volume difference in alcoholic smokers compared to alcoholic non-smokers was found in the left parietal lobe and this was only noted when the threshold for significance was lowered to p < 0.05, for all other comparisons significance was set at p < 0.01.

### 3.6 Cognitive and clinical laboratory results

There were no differences in age or BMI in the ‘pure’ alcoholics and polysubstance abusing alcoholics and healthy controls. Healthy controls had significantly more years of education than either alcoholic group. ‘Pure’ alcoholics displayed higher transferrin values than polysubstance abusing alcoholics. Healthy controls had significantly lower liver enzyme functions: alanine aminotransferase (ALT), aspartate aminotransferase (AST), Gamma-glutamyl transpeptidase (GGT), and mean corpuscular volume (MCV). The groups did not differ on albumin or vitamin B12 values. Healthy controls scored significantly higher on scaled vocabulary, scaled block design, and Trails B performance than either alcoholic group. Controls also had significantly better performance on the Buschke total trials, Stroop color word score, and Trails A score than the polysubstance abusing alcoholic population. ‘Pure’ alcoholics had a higher scaled vocabulary score than the polysubstance abusing alcoholics (Table 3).

**Table 3:** Cognitive and laboratory results.

Characteristic of participants	‘Pure’ alcoholics N = 37		Polysubstance abusing alcoholics N = 93		Healthy controls N = 69	
	Mean	SD	Mean	SD	Mean	SD
Vocabulary Score^a^, ^c^	10.9	0.5	9.7	0.3	12.5	0.3
Block Score^a^	8.9	0.6	8.9	0.3	10.9	0.4
ALT-GPT^a^	53.3	51.5	50.0	49.6	25.7	23.7
AST-GOT^a^	57.5	59.4	55.2	63.8	24.5	8.7
Albumin	4.4	0.4	4.2	0.4	4.4	0.4
GGT^a^	139.4	160.6	116	183.9	19.8	9.9
MCV^a^	93.9	6.7	93.8	5.8	89.5	5.5
Transferrin^c^	273.1	50.0	251.8	37.8	N/A	N/A
Vitamin B12	491.3	228.3	486.7	246.8	N/A	N/A
Buschke Total Trials^b^	7.47	1.0	8.0	1.8	7.09	0.3
Stroop: Color Word^b^	48.3	6.9	44.9	8.5	51.65	1.4
Trails A (s)^b^	36.1	13.9	33.2	10.8	29.53	1.5
Trails B (s)^a^	77.4	36.8	79.0	32.5	57.98	3.8

a Significant (p < 0.05) difference between alcoholics and controls.

b Significant (p < 0.05) difference between controls and poly.

c Significant (p < 0.05) difference between poly and pure.

## 4 Discussion

Although under this study we cannot rule out preexisting reduced gray matter volume for our alcoholic subjects, consistent with the literature we found large reduced gray matter volume in mesial region of the frontal lobes among alcoholic inpatients compared to healthy non-alcoholics. We also found regions of gray matter alterations present in alcoholics who did not meet lifetime diagnostic criteria for any substance abuse or dependence other than alcohol (‘pure’ alcoholics) that were not present in alcoholics who met DSM IV criteria for other substance abuse or dependence. This alcohol specific gray matter alteration was similar in location to the pattern characteristic of Wernicke–Korsakoff Syndrome.

When we compared polysubstance abusing alcoholics with controls we found that polysubstance abusing alcoholics on the average had 45.71 ml less cortical volume. This was similar to the average of 51.51 ml of significant cortical volume deficit observed in the ‘pure’ alcoholics. However, this similarity in the magnitude of cortical volume differences contrasted dramatically with the difference between alcoholic groups in terms of subcortical volume; the ‘pure’ alcoholics had 8.49 ml of significant subcortical gray matter volume reduction compared to the controls (see Table 2), while the polysubstance abusing alcoholics had no significant subcortical volume difference.

The differences we found between ‘pure’ and polysubstance abusing alcoholics match the pattern of gray matter damage classically described in Wernicke's Encephalopathy (WE). Acute WE is characterized by damage to the gray matter of the mammillary bodies, bilateral thalamus, periaqueductal, and periventricular gray matter, and inferior and superior colliculi (Sullivan and Pfefferbaum, 2009). The gray matter alterations found in alcoholics with only alcohol dependence relative to polysubstance abusers was in the mammillary bodies, bilateral thalamus, and dorsal midbrain (Fig. 2). WE has been considered secondary to a deficit of thiamine believed to occur episodically or chronically among alcoholics. Although we did not measure thiamine in our sample, the alcohol only dependent subjects did not display a significant difference in Body Mass Index (BMI), alanine transaminase (ALT), aspartate transaminase (AST), albumin, or Vitamin B12, compared to alcoholic polysubstance abusers. ‘Pure’ alcoholics displayed significantly higher transferrin values, consistent with a slightly better diet than that of the polysubstance abusing alcoholics. This suggests that nutrition deficits and hepatic function (at least at time of admission) do not explain the difference in subcortical volume between the two groups. However, it is possible that some of the alcoholic subjects had episodes of thiamine deficiency in the past.

In addition, WE is characterized by changes in mental status and a deficit in episodic memory, and our ‘pure’ alcoholics did not perform worse than the alcoholics with polysubstance abuse on the Buchske selective reminding memory task, a Trail Making Test (TMT), or block design IQ estimates. ‘Pure’ alcoholics displayed a significantly higher verbal IQ and a higher Stroop color-word score.

Obviously our alcohol only alcoholics did not suffer from classic Wernicke–Korsakoff's syndrome. However, recently Pitel et al. (2012) presented evidence that a neuropathological continuum exists from uncomplicated, cognitively intact alcoholism, to Korsakoff's Syndrome (KS). The KS patients and uncomplicated alcoholics showed similar gray matter alterations bilaterally in the orbitofrontal cortex, the insula, medial temporal lobe, thalami, hypothalamus, and the cerebellum. The thalami, mammillary bodies, and corpus callosum all showed graded volume loss, with KS having the most extensive volume loss, followed by uncomplicated alcoholics. The left thalamic radiation was the only brain region to show exclusive volume loss in KS patients. (Pitel et al., 2012). Other investigators have also suggested that there is a continuum of the neurotoxic effects of alcohol from uncomplicated alcoholism to KS (Blansjaar et al., 1992, Sullivan and Pfefferbaum, 2009).

There are several possible reasons that the alcoholics with polysubstance abuse display more gray matter volume than the alcoholics with only alcohol dependence. Although both groups of alcoholics reported similar history of alcohol use, it is possible that the polysubstance abusing alcoholics actually consumed less alcohol (despite their self report that they consumed similar amounts compared to the ‘pure’ alcoholics), perhaps because they had other ways of achieving an intoxicated state. In addition, it is possible that the pattern of alcohol use and diet in general differs between ‘pure’ and polysubstance abusing alcoholics. Another possibility involves a neuroprotective action mediated through the anti-inflammatory effects of cannabinoids. Several studies have posited a role for pro-inflammatory cytokines in alcohol related brain atrophy (Crews et al., 2006, Valles et al., 2004). Cannabinoids acting at the CB_2_ receptor have been shown to possess anti-inflammatory effects, through the suppression of pro-inflammatory cytokines and chemokines (O'Sullivan and Kendall, 2010). If a neuroprotective effect of cannabinoids is in part responsible for the absence of thalamic and midbrain volume loss among the polysubstance abusing alcoholics, then future studies might test this hypothesis by specifically comparing cannabis with non-cannabis abusing alcoholics. There is a final possibility that polysubstance abusers who use stimulants may have gray matter differences that are masked by inflammation caused by stimulant use. Striatal enlargements have been seen in methamphetamine and cocaine users (Chang et al., 2007, Mackey and Paulus, 2013). It is possible that these users have decreased gray matter caused by alcohol use and concurrent increases in gray matter caused by stimulant use. However, volume decreases have also been found in stimulant users and the enlargements seem to be limited to the striatum (Mackey and Paulus, 2013). The differences seen in the ‘pure’ alcoholics relative to the polysubstance abusing alcoholics are located in the diencephalon and midbrain and therefore it is less likely that stimulant enlargement is masking alcohol induced gray matter differences in the polysubstance stimulant abusing population.

The smaller gray matter volume in cocaine-only polysubstance alcoholics may be explained by the large number of cannabis users in the comparison polysubstance group (n = 64). The cannabinoids may be acting in an anti-inflammatory manner and protecting that group from this gray matter deficit. Additionally, comparisons of cocaine-only polysubstance abusers with polysubstance abusing groups (one comparison including and the other comparison excluding amphetamine/stimulant abusers) indicate that the stimulants do not play any compensatory role, hiding gray matter loss associated with alcohol use in the polysubstance abusing group. In the comparison excluding amphetamine/stimulant users, the number of alcoholics with polysubstance abuse who did not use cocaine or amphetamine/stimulant was too small to make any statistically meaningful conclusions. However, it did indicate no differences between polysubstance abusing alcoholics with cocaine-only use and those without any stimulants. The number of polysubstance abusing alcoholics without stimulant use is a limitation and prevents us from conclusively determining effects of such a condition. Further studies are required to investigate the role of stimulants directly.

Although the ‘pure’ and polysubstance abusing alcoholics differed in gray matter damage in the diencephalon and midbrain they had similar gray matter difference compared to controls in the frontal and temporal cerebral cortex. Does this mean that the ‘pure’ alcoholics had more frequent and/or severe episodes of subclinical thiamine deficiency, as has been suggested as an explanation for the subcortical damage observed in non-Wernicke–Korsakoff Syndrome alcoholics (Sullivan and Pfefferbaum, 2009), but the putative episodes of thiamine deficit do not affect cortex? Or does the similarity between the two groups of alcoholics suggest that their cortical damage precedes substance abuse and is not directly related to any specific drug related neurotoxicity? The limitations of a cross-sectional study make it difficult to determine if the cortical differences we have found between all alcoholics and healthy controls are the consequence of an effective reduced thickness or atrophy in the gray matter, or an indirect reflection of a different gyrification pattern that could have preceded substance abuse. Additionally VBM has inherent limitations, specifically regarding the spatial normalization step (Bookstein, 2001). Smoothing and blurring may also influence the gray matter differences found in very small structures such as the hippocampus. This area should be further investigated in comparisons of alcoholics and healthy controls, as it was not found to be a significant area in our pure vs. poly comparison.

The polysubstance abusing alcoholics displayed a slightly worse performance on cognitive tasks relative to healthy controls. Polysubstance abusers had increased total trials of the Buschke memory test, lower Stroop color word scores, and longer Trails A and B trials all relative to healthy controls. ‘Pure’ alcoholics did not differ significantly from healthy controls on these measures. The slight cognitive deficits that the polysubstance abusing alcoholics exhibit may be a result of the somewhat greater fronto-cortical gray matter alterations they display. Both alcohol dependent groups show cortical differences, however, the gray matter alterations in the polysubstance group is more concentrated in the mesial frontal lobe, while the ‘pure’ alcoholics display a slightly more diffuse pattern.

Another limiting factor of our study is an inability to fully account for potential effect of individual past Axis I disorders. A good number of alcoholic subjects did have some (in most cases multiple) form of past diagnoses. We performed VBM analysis between all alcoholic subjects with anxiety vs. all alcoholics without anxiety and similarly for cases with depression and PTSD. If any of these psychological conditions were the cause of VBM differences between ‘pure’ alcoholics and those with polysubstance abuse, we would expect to see overlapping regions between these comparisons and the ‘pure’ vs. polysubstance abusing alcoholics comparison. The fact that we did not observe such regions at least partially rules out such interaction. We did observe more gray matter in the three psychiatric populations relative to the non-comorbid alcoholics. Enlarged gray matter in anxiety patients is consistent with recent literature (Etkin et al., 2009, Schienle et al., 2011). The literature on brain volume in major depressive disorder largely supports a reduction in gray matter, although results have been mixed (Bremner et al., 2002, Lange and Irle, 2004, van Tol et al., 2010). Some volumetric studies in PTSD have shown reductions in gray matter (Nardo et al., 2010, Zhang et al., 2011), while others have shown no result (Corbo et al., 2005, Jatzko et al., 2006). Because of the mixed results in the literature and the large overlap in patient groups due to comorbidities, more research on this subject is required. To fully rule out the role of psychiatric comorbidity, new populations of alcoholics without psychiatric diagnoses will need to be studied.

The Wernicke–Korsakoff Syndrome-like results of this study call for further research. It is important to investigate the spectrum from the uncomplicated alcoholic to those with Wernicke–Korsakoff Syndrome and elucidate if and how nutrition deficits are involved in the specific pattern of neurodegeneration. Longitudinal studies following a population of alcoholics, both relapsing and abstinent, might help to illustrate this spectrum of gray matter volume differences. Additional cognitive and memory tests should be performed in order to clarify the effect of the gray matter differences present in the alcoholics with only alcohol dependence.
